# The Effect of Nicotinamide Mononucleotide and Riboside on Skeletal Muscle Mass and Function: A Systematic Review and Meta‐Analysis

**DOI:** 10.1002/jcsm.13799

**Published:** 2025-04-24

**Authors:** Konstantinos Prokopidis, Frank Moriarty, Gülistan Bahat, Joseph McLean, David D. Church, Harnish P. Patel

**Affiliations:** ^1^ Department of Musculoskeletal and Ageing Science, Institute of Life Course and Medical Sciences University of Liverpool Liverpool UK; ^2^ School of Pharmacy and Biomolecular Sciences RCSI University of Medicine and Health Sciences Dublin Ireland; ^3^ Istanbul Medical Faculty, Department of Internal Medicine, Division of Geriatrics Istanbul University Istanbul Türkiye; ^4^ Androlabs London UK; ^5^ Department of Geriatrics University of Arkansas for Medical Sciences Little Rock Arkansas USA; ^6^ NIHR Southampton Biomedical Research Centre University of Southampton Southampton UK; ^7^ Academic Geriatric Medicine University of Southampton Southampton UK

**Keywords:** nicotinamide mononucleotide, nicotinamide riboside, physical performance, sarcopenia, skeletal muscle

## Abstract

**Introduction:**

Sarcopenia is associated with the loss of skeletal muscle function and mass. Nicotinamide precursors, such as nicotinamide mononucleotide (NMN) and nicotinamide riboside (NR), have received attention for their potential to improve NAD^+^ levels and mitigate age‐related sarcopenia in preliminary models, though evidence on their effects in older adults remains inconclusive.

**Methods:**

We searched PubMed, Cochrane Library, Web of Science, and Scopus to identify randomized controlled trials (RCTs), comparing NR or NMN vs. placebo. A random‐effects meta‐analysis was employed to determine their impact on measures of sarcopenia such as skeletal muscle index (SMI), handgrip strength (HGS) and gait speed. A narrative synthesis was used for 5‐time chair stand test (5CST), short physical performance battery (SPPB), timed‐up‐and‐go (TUG), 6‐min walking distance (6MWD), leg and chest press 80% 1RM (repetition maximum) and thigh muscle mass.

**Results:**

Included participants had a mean age range from 60.9 to 83 years. NMN supplementation showed no significant effects on SMI (*n* = 3; mean difference (MD): −0.42, 95% confidence interval (CI): −0.99 – 0.14, *I* ^2^ = 63%, *p* = 0.14), HGS (One study estimating left grip; *n* = 5; MD: 0.61, 95%CI: −0.89 – 2.10, *I* ^2^ = 0%, *p* = 0.42; One study estimating right grip; *n* = 5; MD: 0.45, 95%CI: −1.06 – 1.96, *I* ^2^ = 0%, *p* = 0.56), gait speed (*n* = 4; MD: ‐0.01, 95%CI: −0.08 – 0.06, *I* ^2^ = 0%, *p* = 0.79), or 5CST (*n* = 2; MD: ‐0.21, 95%CI: −0.70 – 0.29, *I* ^2^ = 11%, *p* = 0.41). Additionally, our narrative synthesis showed that NMN did not improve knee extension strength, SPPB, or thigh muscle mass. NR supplementation was associated with a longer 6MWD among individuals with peripheral artery disease. However, lower scores in the SPPB and slower 5CST were observed in those with mild cognitive impairment.

**Conclusions:**

Current evidence does not support NMN and NR supplementation for preserving muscle mass and function in adults with mean age of over 60 years. Future research should explore supplementation dosage, NAD^+^ baseline deficiency, and combined interventions.

## 1 Introduction

Sarcopenia is characterized by a gradual but progressive decline of skeletal muscle function and mass [1], and is associated with a range adverse outcomes such as falls, physical frailty, institutionalisation, and increased mortality [2]. While primary sarcopenia is associated with the ageing process, in secondary sarcopenia, multiple factors, including chronic diseases, physical inactivity, hormonal imbalance and malnutrition amplify this age associated phenomenon [3]. These factors contribute to an increase in circulating pro‐inflammatory cytokines [4], oxidative stress [5], dysregulated mitochondrial networks and target genes [6, 7] and anabolic resistance [8], which in combination with loss of motor neurons and myofibre atrophy [9], lead to the decline in skeletal muscle strength and mass.

Currently, non‐pharmacological interventions for the management of sarcopenia primarily involve resistance exercise [10] and sufficient energy and protein intake [11] and potentially incorporation of essential amino acids [12], creatine monohydrate [13] and omega‐3 fatty acids [14]. Although pharmacological interventions such as myostatin inhibitors [15], anabolic steroids [16] and selective androgen receptor modulators [17], have been promising targets of research, none of the interventions have consistently shown to have beneficial effects on muscle function and have been associated with a range of adverse effects that limit their use [18].

Nicotinamide adenine dinucleotide (NAD^+^), a coenzyme involved in energy metabolism, declines during ageing and is linked with reduced DNA repair and immune function that contribute to cellular senescence [19]. Nicotinamide precursors, such as nicotinamide mononucleotide (NMN) and nicotinamide riboside (NR), have garnered attention for their potential ‘anti‐ageing’ effects via enhancement of NAD^+^ levels. Specifically, these compounds may aid in restoring NAD^+^ levels, promoting improved mitochondrial function [20, 21] and glucose profile [22, 23]. There is evidence that improved NAD^+^ levels decrease circulating inflammatory cytokines [24], and improve muscle strength in mice models [25], suggesting therapeutic potential against age‐related sarcopenia. However, research on their effects pertinent to physical performance and muscle strength measures in older adults is controversial [26]. A previous systematic review concluded that there insufficient evidence to determine whether supplementation with NAD^+^ precursors can enhance physical performance or reduce frailty [27]. It is worth mentioning however, that the study's conclusions were constrained by the diverse nature of the study populations, varied methodologies and different outcome measures. More recently, another systematic review suggested a non‐significant improvement in handgrip strength (HGS) and skeletal muscle index (SMI) following NMN supplementation [28]. Considering the significance of these supplements on ageing populations and the inconsistencies surrounding its application in the field of sarcopenia, the purpose of this review was to quantitatively the impact of NMN and NR on muscle mass, strength and physical performance among older adults.

## 2 Methods

This systematic review and meta‐analysis was performed in accordance with the Preferred Reporting Items for Systematic Reviews and Meta‐Analyses (PRISMA) guidelines [29]. The protocol is registered in the International Prospective Register of Systematic Reviews (PROSPERO) (CRD42024558548).

### 2.1 Search Strategy

Two reviewers (KP and JM) searched PubMed, Cochrane Library, Scopus and Web of Science independently from inception until August 2024. The full search strategy and the search terms used are described in Table S1. Discrepancies in the literature search process were resolved by a third investigator.

### 2.2 Inclusion and Exclusion Criteria

Studies were included based on the following criteria: (i) randomized controlled trials (RCTs); (ii) adults with mean age of 60 years and above irrespective of health status; The population of interest for this review was older adults > 60 years, considering that a strict definition for ‘older’ is difficult due to the differences in the rate of biological ageing between individuals. Hence, this cut‐off was used to help standardize the search across databases; and (iii) the intervention group received NAD^+^ precursors, such as NMN or NR; (iv) comparator group receiving placebo; (v) outcomes of interest related to skeletal muscle index, muscle strength and physical performance without any restrictions on the type of measurements. Published articles were excluded if they (i) included participants with mean age below 60 years of age; (ii) were reviews, letters, animal experiments or commentaries; and (iii) were not published as a full text.

The PICOS framework included:

Population: adults with mean age of 60 years and above irrespective of health status.

Intervention: NMN or NR.

Comparator: Placebo.

Outcomes: Primary ➔ SMI, HGS, GS; Secondary ➔ 6‐min walking distance (6MWD), 5‐time chair stand test (5CST), short physical performance battery (SPPB), timed‐up‐and‐go (TUG), leg and chest press 1RM (repetition maximum) and thigh muscle mass.

### 2.3 Data Extraction and Risk of Bias

Two authors (KP and JM) screened and extracted data independently, which included name of first author, date of publication, participant sample size, sex and age, type of NAD^+^ precursor, its dose and duration, endpoint measurements and whether dietary intake was considered. Disagreements between authors were resolved by an independent reviewer.

### 2.4 Risk of Bias and Quality of Studies

The risk of bias of the included studies was assessed using the Risk‐of‐Bias 2 (RoB2) tool [30] and performed by two reviewers (FM and JM). RoB2 is a comprehensive tool used to assess bias in RCTs based on the following domains: (i) randomization process; (ii) deviations from intended interventions; (iii) missing outcome data; (iv) measurement of the outcome; and (v) selection of the reported result. According to the scoring system, study bias was defined as ‘high’, ‘some concerns’ or ‘low’ [31]. In addition, assessment to evaluate the methodological quality of the included studies was performed through PEDro by two independent reviewers (KP and FM) to further measure the validity of the RCTs in this study.

### 2.5 Statistical Analysis

Quantitative data were analysed as continuous variables, with mean differences calculated by comparing changes in outcomes from baseline to follow‐up between groups. When measurement units varied and could not be standardized for inclusion in the analysis, standardized mean differences were used. For studies that did not report numerical data, graphical values were extracted using WebPlotDigitizer software. Missing standard deviations for changes between baseline and follow‐up outcomes were estimated using available confidence intervals, standard errors, *t*‐values, *p*‐values, or by deriving a correlation coefficient from a similar study's standard deviation, or where none of these were possible, using a conservative value of 0.5. Statistical heterogeneity across studies was evaluated through the overlap of their 95% confidence intervals and measured using Cochran's Q (chi‐square test) and *I* ^2^. The classification of data as moderately heterogeneous was based on *I* ^2^ from 50% to 74.9% and highly heterogeneous from 75% and above [32]. Statistical significance was assessed using the random effects model and inverse‐variance method. Furthermore, sensitivity analyses were performed to evaluate the robustness of reported statistical results by discounting the effect of studies with high risk of bias and studies with comorbidities (i.e., Type 2 diabetes). Additionally, since one study [33] measured the strength of the left and right grip separately without identifying the dominant hand, the primary analysis was conducted two times, treating each value independently. The meta‐analysis was conducted using Review Manager (RevMan 5.4.1) software.

## 3 Results

### 3.1 Search Results and Descriptives

**FIGURE 1:**
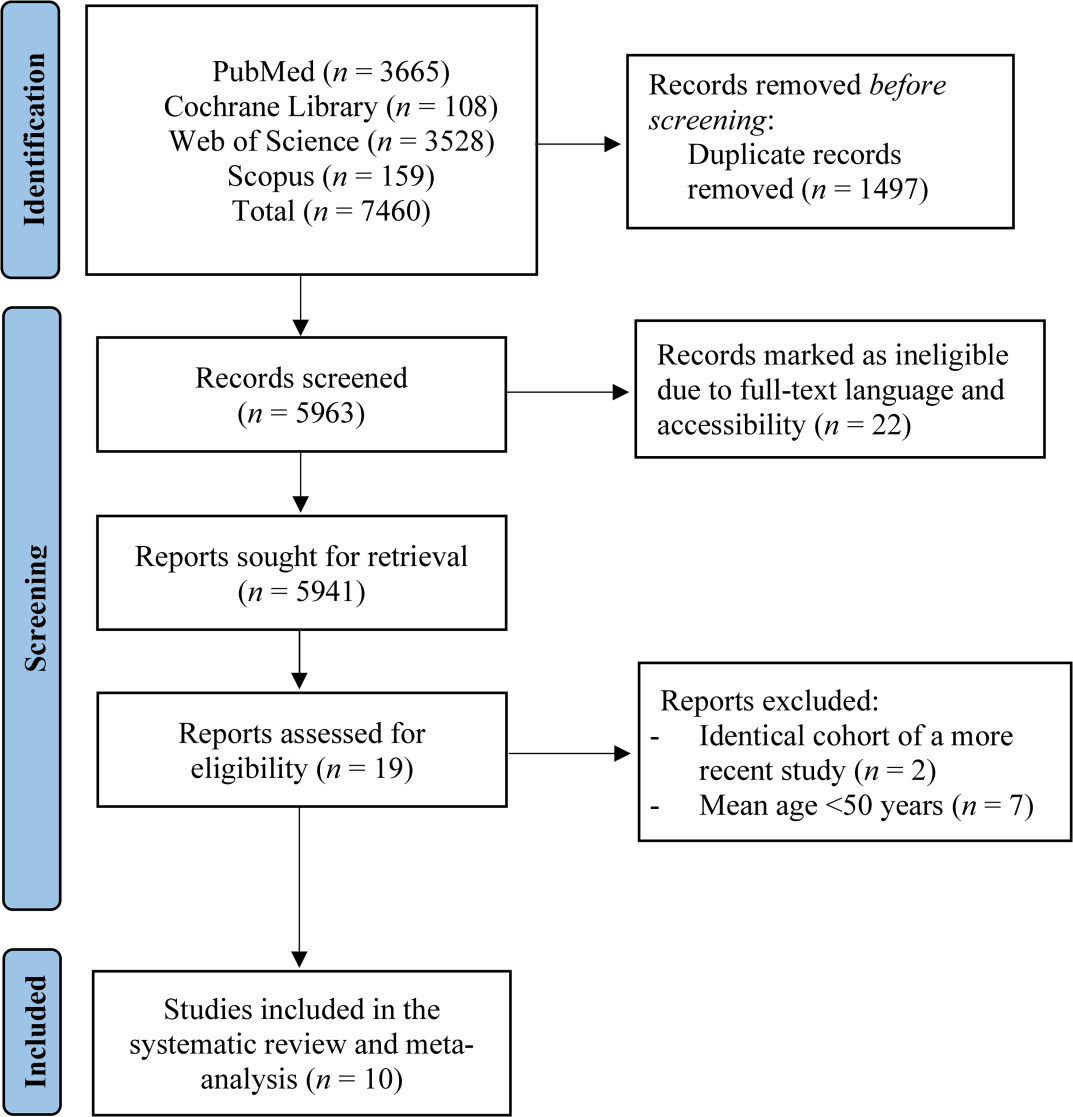
Flowchart of the employed search strategy.

The initial literature search provided 7460 publications. Following the exclusion of duplicates and non‐relevant studies based on titles and abstracts, 19 full texts were identified as potentially eligible for inclusion in the systematic review and meta‐analysis. Of these 19 studies, seven studies included participants with mean age below 60 years of age [34, 35, 36, 37, 38, 39, 40], two studies [41, 42] had an identical cohort of a more recent study that met our inclusion criteria. In total, 10 studies were included in the systematic review and meta‐analysis (Figure 1), from which, six used nicotinamide mononucleotide [33, 43, 44, 45, 46, 47] and four used nicotinamide riboside [48, 49, 50, 51]. The mean age range of the included participants was 60.9–83 years and their mean body mass index between 22.3 and 33.7 kg/m^2^. Treatment dosage was between 250 to 2000 mg/day and treatment duration between 3 and 24 weeks among studies. Characteristics of included studies are outlined in Table 1.

**TABLE 1:** Study and participant characteristics of the included studies. Data are expressed as mean (standard deviation).

Study, year	Health status	NAD^+^ precursor	Intervention			Placebo			Dose and duration	Endpoint	Dietary intake assessment (yes/no)	Physical activity control (yes/no)
			*n* (M/F)	Age (SD)	BMI (kg/m^2^)	*n* (M/F)	Age (SD)	BMI (kg/m^2^)				
Akasaka et al. 2022	Type 2 diabetes	Nicotinamide mononucleotide	7 (7/0)	83 (6.7)	22.3	7 (7/0)	79.3 (6)	23.7	250 mg/d for 24 weeks	HGS, Gait speed, 5CST, SMI, Knee extension	No	No
Kim et al. 2022 (antemeridian)	Community‐dwelling	Nicotinamide mononucleotide	26	72.2 (5.1)	22.9 (2.5)	27 (9/18)	72.5 (4.6)	22.3 (3.2)	250 mg/d for 12 weeks	HGS, 5CST, Gait speed, Timed up and go	No	No
Kim et al. 2022 (postmeridian)	Community‐dwelling	Nicotinamide mononucleotide	27 (9/18)	72.8 (4.3)	23.4 (2.8)	27 (9/18)	73 (4.7)	22.4 (2.2)	250 mg/d for 12 weeks	HGS, 5CST, Gait speed, Timed up and go	No	No
Morifuji et al. 2024	Community‐dwelling	Nicotinamide mononucleotide	30 (18/12)	69 (3)	22.4 (2.6)	29	69 (3)	22.6 (3.6)	250 mg/d for 12 weeks	HGS, SPPB, Gait speed, SMI	No	No Excluded if: daily exercise habit
Igarashi et al. 2022	Community‐dwelling	Nicotinamide mononucleotide	10 (10/0)	71.1 (3.9)	24.1 (1.4)	10 (10/0)	71.8 (6.1)	24.5 (1.4)	250 mg/d for 12 weeks	HGS, 30CST, Gait speed, SMI	No	No Excluded if: daily exercise for at least 1 h for a minimum of 6 months continuously
Yoshino et al. 2021	Prediabetes and overweight	Nicotinamide mononucleotide	13 (0/13)	62 (4)	33.7 (1.4)	12 (0/12)	61 (5)	33.4 (1.0)	250 mg/d for 10 weeks	HGS, FFM	No	No
Pencina et al. 2023	Obesity	Nicotinamide mononucleotide	21 (11/10)	60.9 (8.9)	29.1 (3.57)	9 (5/4)	64.3 (7.6)	29.5 (3.8)	2000 mg/d for 4 weeks	Chest press 1RM, Chest press reps to failure, Leg press 1RM, Leg press reps to failure, Thigh muscle mass	No	Yes
Elhassan et al. 2019	Community‐dwelling	Nicotinamide riboside	12 (12/0)	75 (5)	Median for all: 26.6	12 (12/0)	75 (5)	Median for all: 26.6	1 g/d for 3 weeks	HGS	No	No
Orr et al. 2024	Mild cognitive impairment	Nicotinamide riboside	10 (5/5)	77 (10.3)	28.6 (4.8)	10 (2/8)	76.1 (16.3)	26.7 (3.6)	1 g/d for 10 weeks	HGS, Gait speed, 5CST, SPPB	No	No
Martens et al. 2018	Community‐dwelling	Nicotinamide riboside	12 (6/6)	65 (7)	24 (3)	12 (5/7)	65 (7)	23 (4)	1 g/d for 6 weeks	HGS, Gait speed, 6MWD, 5CST	Yes	Yes
McDermott et al. 2024	Peripheral artery disease	Nicotinamide riboside	24	73.2 (10)	28.7 (3.6)	24	69.7 (8.3)	30.4 (6.4)	500 mg/d for 12 weeks	6MWD	No	Yes

Abbrevation: 1RM, repetition maximum; 5CST, 5‐time chair stand test; 30CST, 30‐s chair stand test; 6MWD, 6‐min walking distance; BMI, body mass index; FFM, fat free mass; HGS, Handgrip strength; SMI, Skeletal Muscle Index; SPPB, Short Physical Performance Battery.

### 3.2 Effects of Nicotinamide Mononucleotide on Skeletal Muscle Index, Handgrip Strength, Gait Speed and 5‐Time Chair Stand Test

**FIGURE 2:**
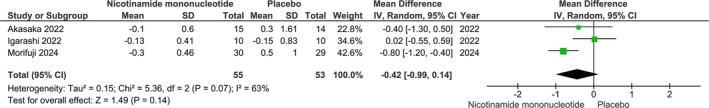
Effect of nicotinamide mononucleotide vs. placebo on skeletal muscle index (kg/m^2^).

No significant changes were observed on SMI (kg/m^2^) following NMN supplementation (*k* = 3; MD: −0.42, 95% CI: −0.99 to 0.14, *I* ^2^ = 63%, *p* = 0.14) (Figure 2). Additionally, considering that the study by Igarashi et al. (2022) [33] used left and right HGS (kg) measurements among participants separately, we conducted our main analysis twice. In both cases, we found no significant differences between NMN and placebo (Addition of one study estimating left grip; *k* = 5; MD: 0.61, 95% CI: −0.89 to 2.10, *I* ^2^ = 0%, *p* = 0.42, Figure S1; Addition of one study estimating right grip; *k* = 5; MD: 0.45, 95% CI: −1.06 to 1.96, *I* ^2^ = 0%, *p* = 0.56, Figure S2). Furthermore, we explored the effects of NMN vs. placebo on HGS exclusively in community‐dwelling adults through a sensitivity analysis, by excluding two studies for which individuals with Type 2 diabetes [44] and prediabetes [43] were included in the main analysis. Similar to our main analyses, no statistically significant differences between groups were found (Addition of one study estimating left grip; *k* = 3; MD: 0.65, 95% CI: −1.27 to 2.57, *I* ^2^ = 0%, *p* = 0.51, Figure S3; Addition of one study estimating right grip; *k* = 3; MD: 0.39, 95% CI: −1.57 to 2.34, *I* ^2^ = 0%, *p* = 0.70, Figure S4). Finally, when we excluded one study due to high risk of bias, results remained statistically insignificant (Addition of one study estimating left grip; *k* = 4; MD: 0.95, 95% CI: −0.66 to 2.57, *I* ^2^ = 0%, *p* = 0.25, Figure S5; Addition of one study estimating right grip; *k* = 4; MD: 0.78, 95%CI: −0.86 to 2.41, *I* ^2^ = 0%, *p* = 0.35, Figure S6).

**FIGURE 3:**
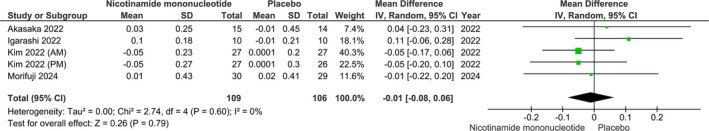
Effect of nicotinamide mononucleotide vs. placebo on gait speed (m/s).

**FIGURE 4:**
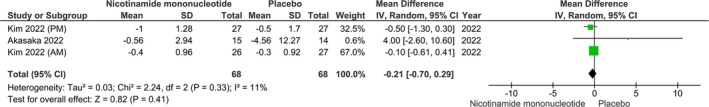
Effect of nicotinamide mononucleotide vs. placebo on 5‐time chair stand test (seconds).

No significant changes between groups were observed in relation to gait speed (m/s) (*k* = 4; MD: −0.01, 95% CI: −0.08 to 0.06, *I* ^2^ = 0%, *p* = 0.79) (Figure 3) nor when a study that included patients with type 2 diabetes was omitted (*k* = 3; MD: −0.01, 95% CI: −0.09 to 0.06, *I* ^2^ = 0%, *p* = 0.72) (Figure S7). Regarding 5CST (seconds), no changes between groups were shown (*k* = 2; MD: −0.21, 95% CI: −0.70 to 0.29, *I* ^2^ = 11%, *p* = 0.41) (Figure 4).

### 3.3 Narrative Synthesis

No statistical changes were observed in one study [33] that studied how many repetitions participants performed during a 30‐s CST, irrespective of treatment duration (after 6 or 12 weeks; 12 weeks: NMN ➔ Δ = 1.5 ± 1.7 repetitions vs. Placebo ➔ Δ = 0.5 ± 3.7 repetitions, *p* = 0.31).

### 3.4 Effects of Nicotinamide Mononucleotide on Other Sarcopenia‐Related Measures

#### 3.4.1 Narrative Synthesis

Other measures examined but not included in our meta‐analysis due to lack of studies, were knee extension strength, SPPB, TUG, chest press 1RM and to failure (80% of 1RM), leg press 1RM and to failure (80% of 1RM) and thigh muscle mass.

Median (interquartile range) changes were insignificant in terms of knee extension following 24 weeks of 250 mg/d NMN supplementation (NMN ➔ Δ = 1.7 (−2.04, 5.36) kg; Placebo ➔ Δ = −0.25 (−3.83, 3.32) kg, *p* = 0.38) [44]. Moreover, no changes were observed in SPPB between NMN (250 mg/d for 12 weeks) and placebo (NMN ➔ 11.9 ± 0.3 to 11.8 ± 0.6 vs. Placebo ➔ 11.7 ± 0.6 to 11.7 ± 0.8, *p* = 0.82) [46]. NMN supplementation of 2000 mg/d for 4 weeks led to no Δ changes in relation to chest press 1RM (*p* = 0.35), chest press to failure (*p* = 0.15), or leg press 1RM (*p* = 0.07), albeit a statistical change on leg press to failure was demonstrated, for which, the placebo group performed more repetitions (*p* = 0.02). Interestingly, NMN treatment resulted in a modest reduction in thigh muscle mass (−70 (−13, −100) g), whereas the placebo group exhibited a slight increase in thigh muscle mass (0.13 (0.04, 0.24) g). The difference between the groups reached statistical significance (*p* < 0.01) [47].

### 3.5 Effects of Nicotinamide Riboside on Handgrip Strength and 6‐Min Walking Distance

#### 3.5.1 Narrative Synthesis

NR supplementation (1 g/d) may not improve HGS after 3 weeks (*p* = 0.96) or 6 weeks (*p* > 0.05) in community‐dwelling [48], and 10 weeks (*p* = 0.11) in individuals with mild cognitive impairment vs. placebo [49]. Likewise, 6 weeks of NR vs. placebo led to no changes on 6MWD (*p* > 0.05) [50]. However, in individuals with peripheral artery disease, a statistically significant improvement after 3 months (mean Δ = 22.4 m, *p* = 0.03) and 6 months (NR ➔ Δ = 7.0 m vs. Placebo ➔ Δ = −10.6, between group difference: 17.6 m) was observed [51].

### 3.6 Effects of Nicotinamide Riboside on Other Sarcopenia‐Related Measures

In individuals with mild cognitive impairment, NR (1 g/d) led to a statistically significant decrease of the SPPB score vs. placebo after 10 weeks (NR ➔ 10.4 ± 1.51 to 9.5 ± 2.12, *p* = 0.13; Placebo ➔ 8.67 ± 2.12 to 10.11 ± 1.62, *p* = 0.04; Group difference ➔ *p* = 0.01) [49]. In the same study, a similar pattern of results was identified for 5CST (seconds) (NR ➔ 14.47 ± 4.67 to 14.21 ± 2.97, *p* = 0.85; Placebo ➔ 17.54 ± 4.57 to 13.35 ± 2.66, *p* < 0.01; Group difference ➔ *p* = 0.03). On the contrary, no changes were on 5CST (seconds) were observed between groups in community‐dwelling individuals (*p* > 0.05). Likewise, no statistically significant changes between NR and placebo were shown in relation to peak torque of both knee flexion and extension strength (*p* > 0.05) [50].

### 3.7 Risk of Bias and Quality Assessment

Overall, six studies had a low risk of bias assessment; two studies had some concerns, while two other studies were estimated to have an increased risk of bias. A more detailed version of each study is presented in Table 2 for parallel group RCTs and Table 3 for crossover RCTs [43, 50]. Quality assessment using the PEDro scale showed good overall quality of the included RCTs (Table S2).

**TABLE 2:** Risk of bias assessment of the included parallel group randomized controlled trials.

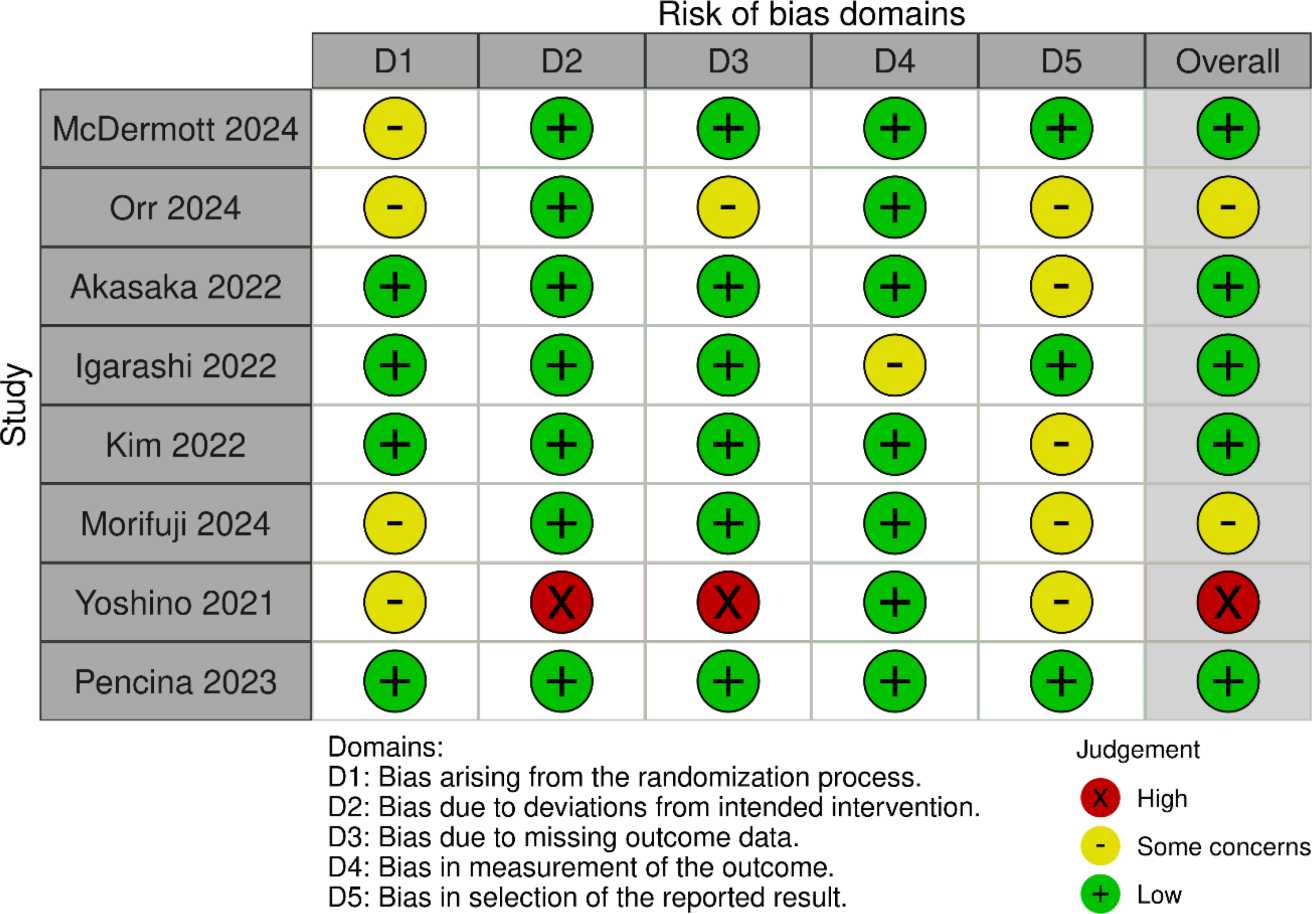

**TABLE 3:** Risk of bias assessment of the included crossover randomized controlled trials.

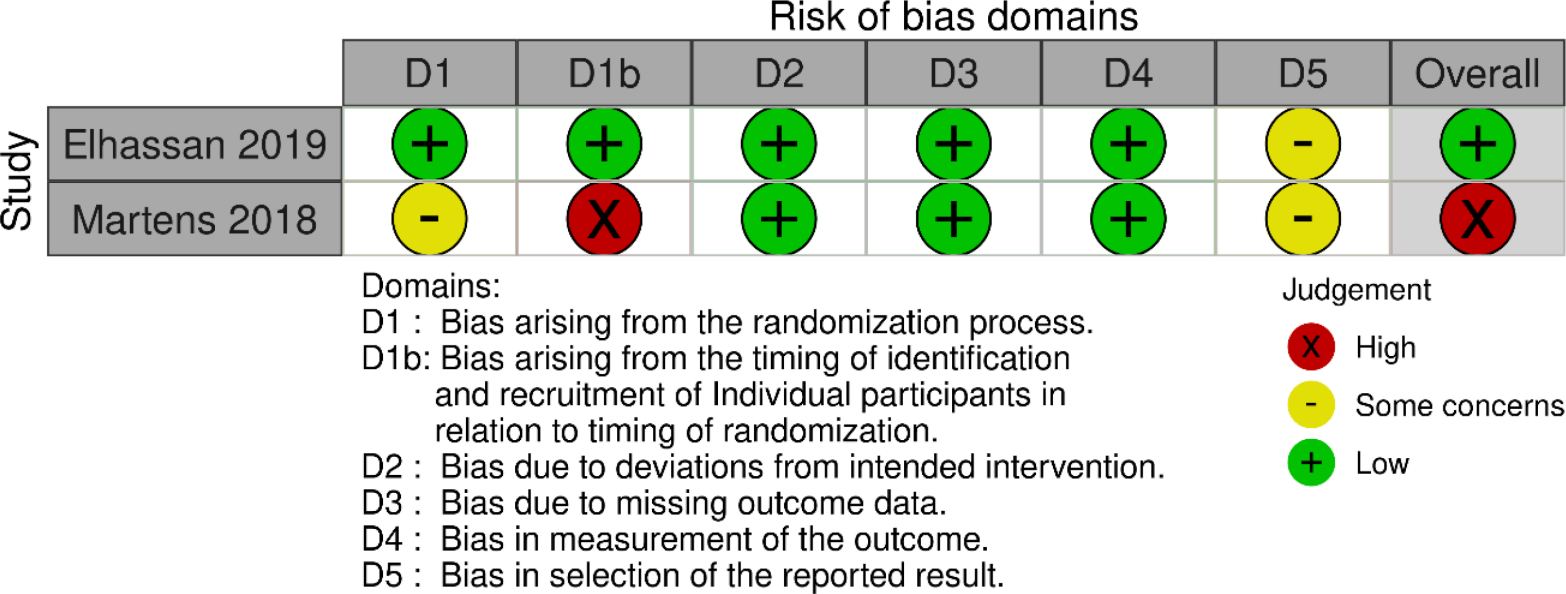

## 4 Discussion

This systematic review and meta‐analysis explored the effects of NMN and NR supplementation on various sarcopenia‐related outcomes, including SMI, HGS, gait speed, and CST. However, the results were largely inconclusive, with no significant changes observed in these measures between those treated with NMN vs. placebo. This was consistent across multiple analyses, including sensitivity analyses that excluded studies with adults accompanied by conditions such as Type 2 diabetes or prediabetes, and studies with high risk of bias. The lack of significant improvement in primary and secondary outcomes following NMN and NR supplementation suggests that this intervention may have a minimal effect on sarcopenia‐related measures in the general older population. Interestingly, reduced performance on SPPB, 5CST and leg press (80% 1RM) to failure were also observed based on findings from one study. These results question utility of NMN and NR as therapeutic agents for the preservation of muscle function in older adults.

Despite positive pre‐clinical findings, our results highlight a negligible effect of both NMN and NR. Further, they highlight the complexity of sarcopenia, a condition that requires multifaceted treatment approaches given the close association with co‐morbid conditions. Despite some evidence of modest improvements in individuals with peripheral artery disease, the overall body of research suggests that the benefits of NR may be limited to specific subgroups rather than the general population. Statistically, NMN was associated with some improvements in leg press performance but decreases in thigh muscle mass. However, these effects were particularly small and clinically insignificant. Moreover, the superior performance of the placebo group in leg press (80% 1RM) to failure raises further questions about the long‐term benefits of these precursors for muscle endurance and strength. Only one study examined an NMN supplementation of dosage of more than 250 mg/d. However, Pencina et al. (2023) utilizing a dosage of 2000 mg/d for 4 weeks in older adults with obesity did not demonstrate any changes regarding chest press 1RM, chest press to failure, leg press 1RM. Interestingly, a change on leg press to failure was demonstrated, for which, the placebo group performed more repetitions. Furthermore, a modest reduction in thigh muscle mass was also shown (−70 [−13, −100] g) compared to placebo (0.13 [0.04, 0.24] g). Increasing the duration of study intervention incorporating such high dosages would help elucidate whether changes in muscle strength and mass persist. The variability in these outcomes may be attributed to several factors, including heterogeneity in study populations, variations in intervention durations and dosages (studies ranging from 3 to 10 weeks), and the absence of key confounding controls, such as monitoring physical activity levels and dietary intake across studies. Nevertheless, more prominent results may be emerged from participants, for whose, NAD^+^ metabolism is severely impaired, such as those with mitochondrial myopathies. For example, 4 months of NR supplementation (1 g/d) resulted in significant improvements in 6MWD, abdominal muscle strength and elbow flexion, and reduced liver fat in adults with mitochondrial myopathy vs. healthy age‐matched controls [52]. However, it is worth noting that: 1) this study was a non‐randomized, open‐label study, which requires further research with a more robust study design, and 2) only the study by Akasaka et al. (2022) had a longer duration than four or more months in our meta‐analysis with a 250 mg/d treatment dosage, that did not show any changes on muscle strength or physical performance in older adults with Type 2 diabetes. Lastly, we may speculate that benefits derived from nicotinamide precursors on mitochondrial morphology may be more applicable in pathways involved in aerobic capacity rather than anaerobic or physical capacity. However, even in this instance, a recent randomized, placebo‐controlled trial in patients with chronic kidney disease who were administered NR (1 g/d) for 6 weeks did not have any improvements in peak oxygen uptake (VO_2peak_) or total work efficiency compared with placebo [21]. Despite promising pre‐clinical outcomes, our findings indicate that NMN and NR supplementation offer minimal benefits for sarcopenia and muscle performance in older adults. However, their potential may be more pronounced in individuals with mitochondrial myopathies and/or impaired NAD^+^ metabolism.

### 4.1 Strengths and Limitations

Our study has several strengths and limitations. We employed a robust approach by identifying studies that used placebo as a control and utilized subgroup and sensitivity analyses to explore more homogeneous groups. As a result of NAD precursor supplementation being a relatively new area, our study pooled data from a small number of available RCTs. In addition, some studies did not account for physical activity. However, assumptions can be made that most older individuals were at least sedentary or were not undergoing resistance training, based on their age and exclusion criteria from some of the included studies (i.e., participants were excluded if they were engaged with daily exercise). In addition, most studies did not control or measure dietary intake at any time, which could have assisted with determining whether higher energy and/or protein intake could have impacted the effectiveness and subsequent results of the intervention and/or the placebo groups. Moreover, the term ‘community‐dwelling’does not imply that those cases are free from Type 2 diabetes, prediabetes or MCI, although it provided us with an additional subgroup analysis based on participant setting. Another limitation may be pertinent to the various dosages employed among studies. For an intervention to demonstrate efficacy, an optimal dose and sufficient duration of application are required. This principle is particularly relevant in conditions such as sarcopenia, where parameters including muscle mass and strength may be relatively easy to lose but significantly more challenging to (re)gain as opposed to younger participants. Finally, considering that muscle mass enhancement is a process that may require a significant duration to be detected, we could not perform subgroup analysis based on different durations of treatment to evaluate the impact of longer‐term trials, quantitatively.

## 5 Conclusions

In conclusion, current evidence does not support the use of NMN and NR as effective interventions for improving muscle function and mass in adults above 60 years old. The inconclusive results observed in this meta‐analysis highlight the need for continued investigation of these compounds' potential role in the regulation of muscle strength and mass and in combination with other therapeutic strategies such as resistance exercise and dietary interventions (i.e., ensuring adequate energy and protein intake). Specific gaps in the research include 1) supplementation in conditions of NAD^+^ deficiency, 2) the combined use of NMN/NR supplementation with exercise training and 3) NMN/NR supplementation in cases where sarcopenia emerges as a secondary condition. Finally, given the strength of pre‐clinical findings and the promising metabolic improvements observed in humans, determining the appropriate length and dosage of supplementation to achieve functional improvements is of primary importance in assessing efficacy.

## Ethics Statement

The authors of this manuscript certify that they comply with the ethical guidelines for authorship and publishing in the Journal of Cachexia, Sarcopenia and Muscle [53].

## Conflicts of Interest

HPP has received lecture fees from Abbott, Pfizer, and HC‐UK conferences outside of the submitted work. The rest of the authors declare that they have conflicts to interest.

## Supporting information


**Figure S1** Effect of nicotinamide mononucleotide vs. placebo on handgrip strength with one study measuring left grip (kg).

**Figure S2** Effect of nicotinamide mononucleotide vs. placebo on handgrip strength with one study measuring right grip (kg).

**Figure S3** Effect of nicotinamide mononucleotide vs. placebo on handgrip strength with one study measuring left grip (kg) while excluding studies with diabetes or prediabetes.

**Figure S4** Effect of nicotinamide mononucleotide vs. placebo on handgrip strength with one study measuring right grip (kg) while excluding studies with diabetes or prediabetes.

**Figure S5** Effect of nicotinamide mononucleotide vs. placebo on handgrip strength with one study measuring left grip (kg) while excluding studies with high risk of bias.

**Figure S6** Effect of nicotinamide mononucleotide vs. placebo on handgrip strength with one study measuring right grip (kg) while excluding studies with high risk of bias.

**Figure S7** Effect of nicotinamide mononucleotide vs. placebo on gait speed (m/s) while excluding studies with diabetes or prediabetes.

**Table S1** Search terms employed in the screening based on title, abstract and keywords in the literature search.

**Table S2** Quality assessment of the included trials using the PEDro scale.

**Data S1** Supplementary Information.

## Data Availability

Data is available upon request.
