# Supplementary figures and images for: The Effect of Nicotinamide Mononucleotide and Riboside on Skeletal Muscle Mass and Function: A Systematic Review and Meta‐Analysis
Source: J Cachexia Sarcopenia Muscle. 2025 Apr 24;16(3):e13799. doi: 10.1002/jcsm.13799 (PMC12022230; doi:10.1002/jcsm.13799)

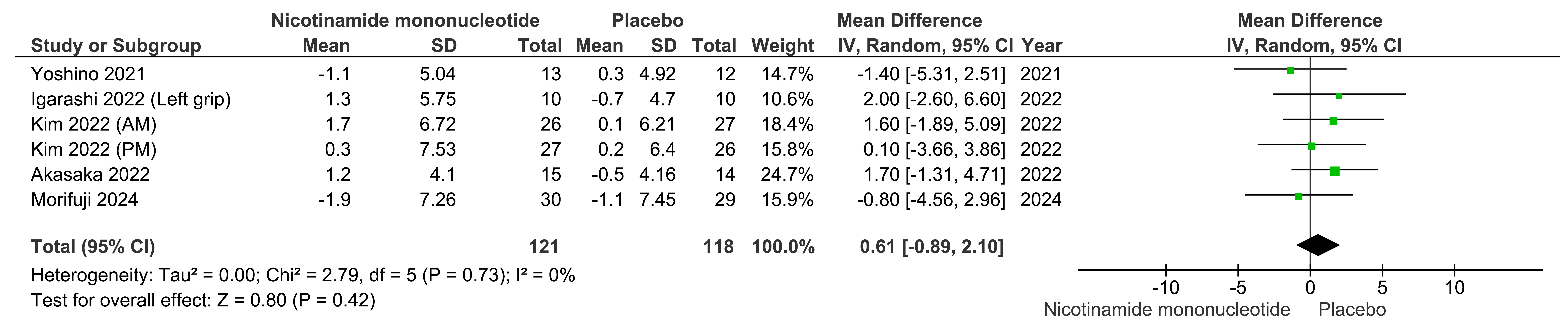

Supplement: Supplementary file 1 — Figure S1 Effect of nicotinamide mononucleotide vs. placebo on handgrip strength with one study measuring left grip (kg). [file JCSM-16-e13799-s010.tiff]

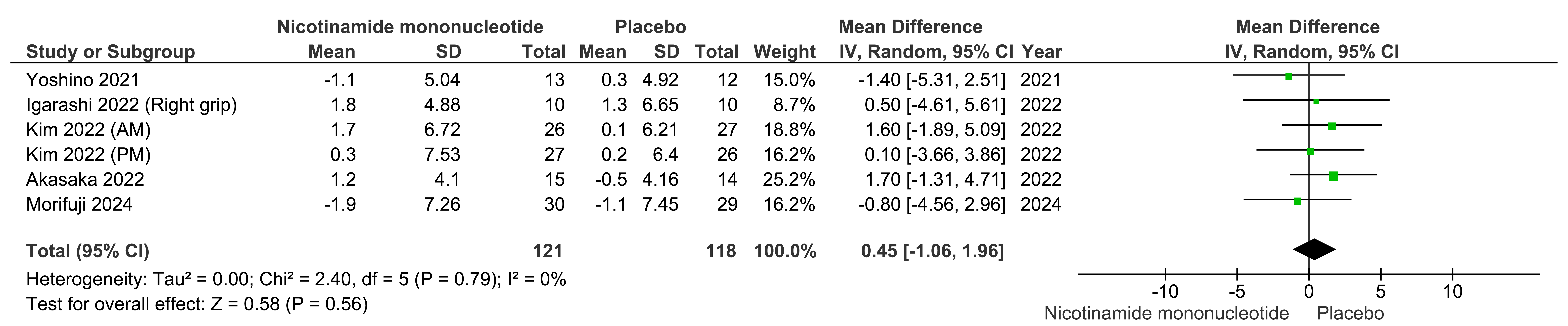

Supplement: Supplementary file 2 — Figure S2 Effect of nicotinamide mononucleotide vs. placebo on handgrip strength with one study measuring right grip (kg). [file JCSM-16-e13799-s007.tiff]

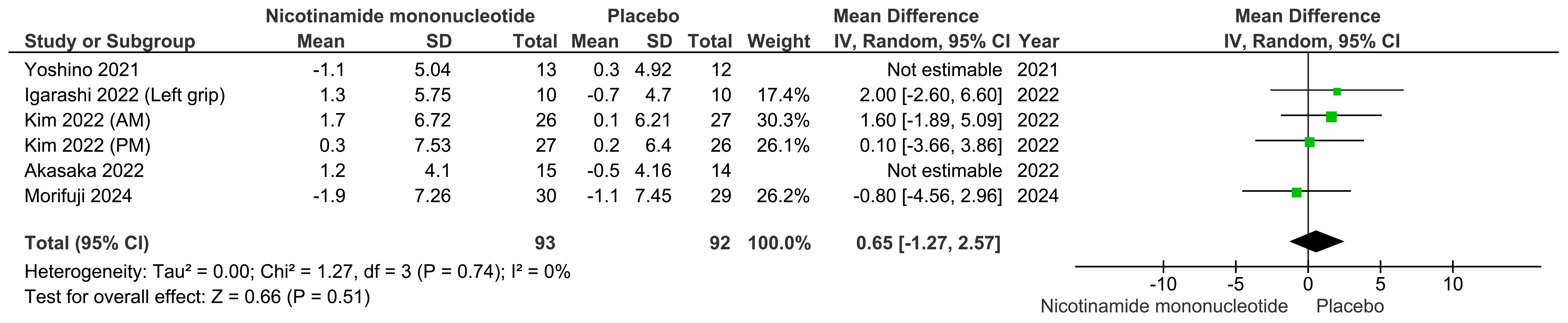

Supplement: Supplementary file 3 — Figure S3 Effect of nicotinamide mononucleotide vs. placebo on handgrip strength with one study measuring left grip (kg) while excluding studies with diabetes or prediabetes. [file JCSM-16-e13799-s009.tiff]

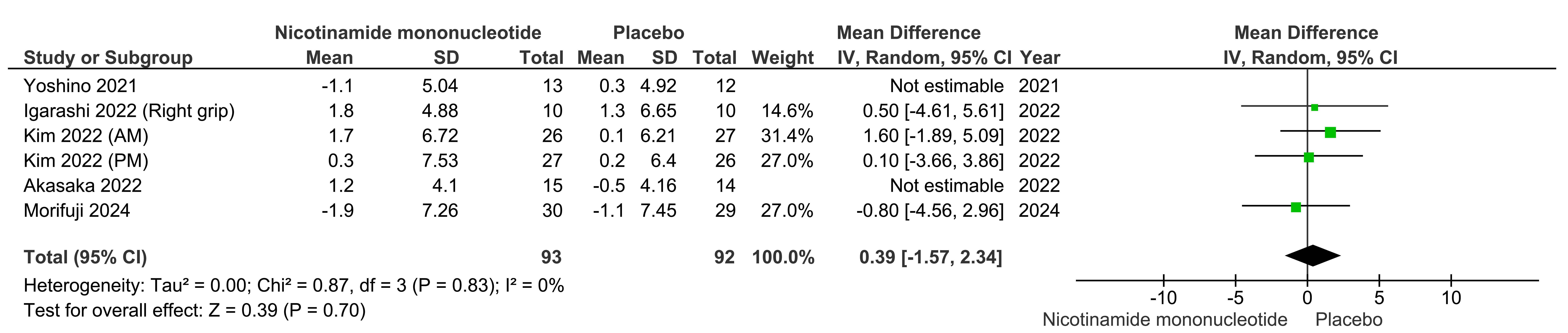

Supplement: Supplementary file 4 — Figure S4 Effect of nicotinamide mononucleotide vs. placebo on handgrip strength with one study measuring right grip (kg) while excluding studies with diabetes or prediabetes. [file JCSM-16-e13799-s006.tiff]

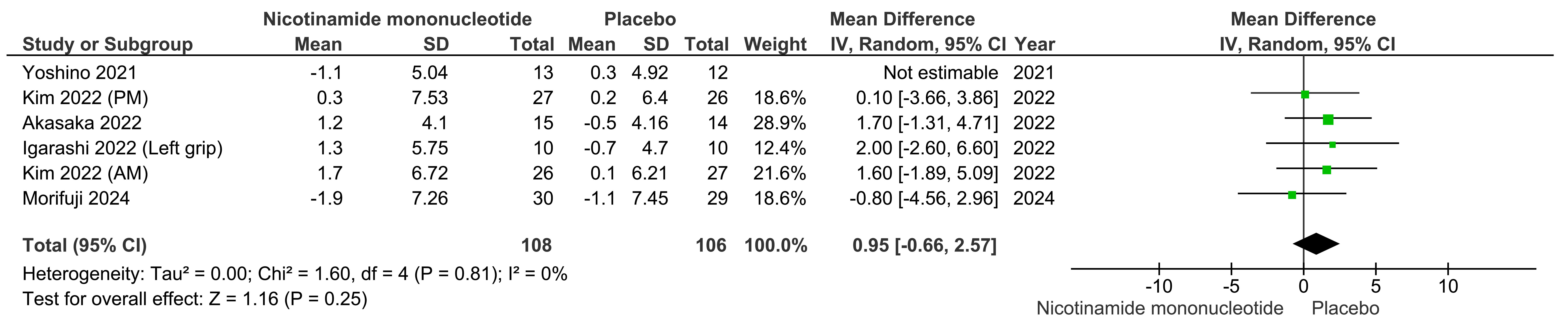

Supplement: Supplementary file 5 — Figure S5 Effect of nicotinamide mononucleotide vs. placebo on handgrip strength with one study measuring left grip (kg) while excluding studies with high risk of bias. [file JCSM-16-e13799-s001.tiff]

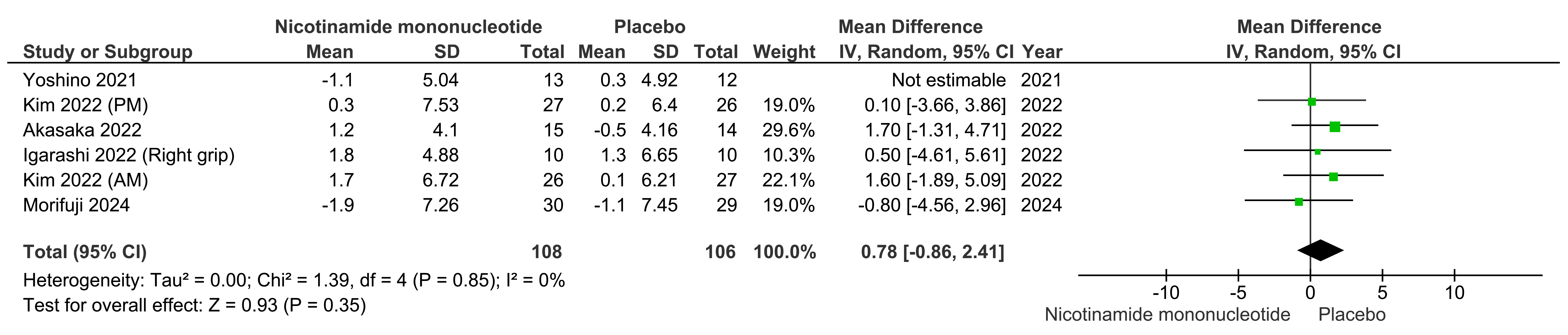

Supplement: Supplementary file 6 — Figure S6 Effect of nicotinamide mononucleotide vs. placebo on handgrip strength with one study measuring right grip (kg) while excluding studies with high risk of bias. [file JCSM-16-e13799-s004.tiff]

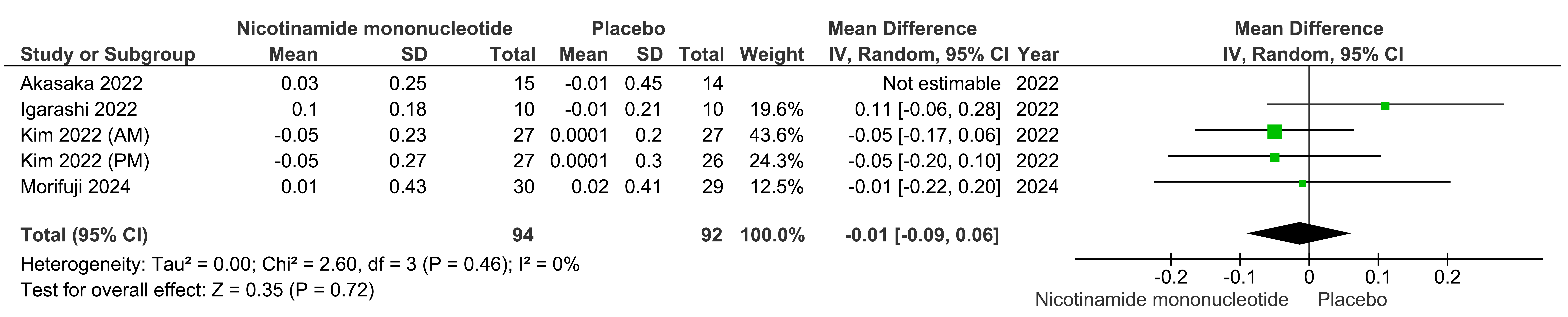

Supplement: Supplementary file 7 — Figure S7 Effect of nicotinamide mononucleotide vs. placebo on gait speed (m/s) while excluding studies with diabetes or prediabetes. [file JCSM-16-e13799-s003.tiff]
