# Supplementary material for: The Effect of Nicotinamide Mononucleotide and Riboside on Skeletal Muscle Mass and Function: A Systematic Review and Meta‐Analysis
Source: J Cachexia Sarcopenia Muscle. 2025 Apr 24;16(3):e13799. doi: 10.1002/jcsm.13799 (PMC12022230; doi:10.1002/jcsm.13799)
Supplement: Supplementary file 8 — Table S1 Search terms employed in the screening based on title, abstract and keywords in the literature search. [file JCSM-16-e13799-s005.docx]

**Table S1.**Search terms employed in the screening based on title, abstract, and keywords in the literature search.

| **Database** | **Search terms** |
| --- | --- |
|  |  |
| PubMed | (“Trigonelline” OR "Uthever" OR "Niagen" OR "Tru Niagen" OR “MIB-626” OR "Niacinamide" OR "nicotinamide riboside" OR  "Nicotinamide Mononucleotide" OR "Nicotinamide Riboside Chloride" OR "β-nicotinamide mononucleotide" OR  "Nicotinamide Riboside Hydrogen Malate" OR "NAD " OR "Nicotinamide Adenine Dinucleotide")  AND (skeletal muscle index OR appendicular lean mass OR skeletal muscle OR muscle strength OR muscle mass OR lean mass  OR muscle protein OR walking speed OR gait speed OR handgrip strength OR grip strength OR lean body mass OR fat free mass  OR muscle mass) |
| Cochrane Library | (“Trigonelline” OR "Uthever" OR "Niagen" OR "Tru Niagen" OR “MIB-626” OR "Niacinamide" OR "nicotinamide riboside" OR  "Nicotinamide Mononucleotide" OR "Nicotinamide Riboside Chloride" OR "β-nicotinamide mononucleotide" OR  "Nicotinamide Riboside Hydrogen Malate" OR "NAD " OR "Nicotinamide Adenine Dinucleotide")  AND (skeletal muscle index OR appendicular lean mass OR skeletal muscle OR muscle strength OR muscle mass OR lean mass  OR muscle protein OR SPPB OR walking speed OR gait speed OR handgrip strength OR grip strength OR lean body mass  OR fat free mass OR muscle mass) |
| Web of Science | ("Niacinamide" OR "nicotinamide riboside" OR "Nicotinamide Mononucleotide" OR "Nicotinamide Riboside Chloride" OR  "β-nicotinamide mononucleotide" OR "Nicotinamide Riboside Hydrogen Malate" OR "NAD " OR "Nicotinamide Adenine Dinucleotide")  AND (skeletal muscle index OR appendicular lean mass OR skeletal muscle OR muscle strength OR muscle mass OR lean mass OR  muscle protein OR SPPB OR walking speed OR gait speed OR handgrip strength OR grip strength OR lean body mass OR  fat free mass OR muscle mass) |
| Scopus | ((nicotinamide AND riboside) OR (nicotinamide AND mononucleotide)) AND ((skeletal AND muscle) OR  (muscle AND strength) OR (muscle AND mass) OR (gait AND speed) OR (handgrip)) |
