# Supplementary material for: The Effect of Nicotinamide Mononucleotide and Riboside on Skeletal Muscle Mass and Function: A Systematic Review and Meta‐Analysis
Source: J Cachexia Sarcopenia Muscle. 2025 Apr 24;16(3):e13799. doi: 10.1002/jcsm.13799 (PMC12022230; doi:10.1002/jcsm.13799)
Supplement: Supplementary file 9 — Table S2 Quality assessment of the included trials using the PEDro scale. [file JCSM-16-e13799-s002.docx]

**Table S2.** Quality assessment of the included trials using the PEDro scale.

| Study | Q1 | Q2 | Q3 | Q4 | Q5 | Q6 | Q7 | Q8 | Q9 | Q10 | Q11 |
| --- | --- | --- | --- | --- | --- | --- | --- | --- | --- | --- | --- |
| Akasaka, 2022 | Yes.  ‘' The inclusion criteria were male patients aged ≥65 years undergoing treatment for type 2 diabetes, reduced grip strength (<26 kg) or  reduced walking speed (<1.0 m/s) and HbA1c level ≥6.5%. The  exclusion criteria included difficulty in independent living, malignant tumours, dementia, severe heart failure, severe hepatic dysfunction, chronic renal failure, haematological diseases, severely  impaired nutritional levels and severely worsened general  condition. (P2;39) | Yes  ‘’ Among these patients, we estimated a maximum of 16 male  patients who met the inclusion criteria, and we equally divided  them into NMN and placebo groups for a simple randomized  controlled study.’’  (P2;39) | Yes  ‘’ The allocation of which was independent of the hospital,  carried out random assignments. It used a computer program to  create a randomized allocation table and label the drugs with  NMN or placebo based on the table. The allocation table was kept  strictly con dential by the secretariat until the end of the follow-  up period. During the study period, the allocation of ce delivered  the study medications, as requested by the hospital. All the study  participants, physicians and laboratory personnel were blinded to  the allocation results.’’  (P2;39) | Yes  (P3;40) | Yes  (P3;40) | Yes  ‘’ The allocation of ce, which was independent of the hospital,  carried out random assignments. It used a computer program to  create a randomized allocation table and label the drugs with  NMN or placebo based on the table. The allocation table was kept  strictly confidential by the secretariat until the end of the follow-  up period. During the study period, the allocation of delivered  the study medications, as requested by the hospital. All the study  participants, physicians and laboratory personnel were blinded to  the allocation results.’’  (P2;39) | Yes  ‘’ The allocation of ce, which was independent of the hospital,  carried out random assignments. It used a computer program to  create a randomized allocation table and label the drugs with  NMN or placebo based on the table. The allocation table was kept  strictly confidential by the secretariat until the end of the follow-  up period. During the study period, the allocation of delivered  the study medications, as requested by the hospital. All the study  participants, physicians and laboratory personnel were blinded to  the allocation results.’’  (P2;39) | Yes, 14/16 (P3;40) | Yes, (P3;40) | Yes, (P4;41 | Yes, table 3 (P5;42) |
| Igarashi, 2022 | Yes, (P8) ‘’ The inclusion criteria were as follows: male, aged more than 65 years, BMI  (in kg/m2) 22–28, nonsmokers, and without any active diseases.  Participants with a history of treatment for malignancy, heart failure, or  myocardial infarction; consuming a prescription medication and/or  supplement that may affect the ndings of clinical research;’’ | yes (p2)  ‘’42 eligible  participants were selected and randomized in a 1:1 ratio into the  two groups. ‘’ | Yes (P8)  ‘’The  allocation to the NMN or placebo group was also managed by C&C  QUALITATIVE RESEARCH INSTITUTE INC until the end of the study. The  participants received oral supplementation of 250 mg of NMN (Mitsubishi  Corporation Life Sciences Limited, Tokyo, Japan) once daily or a placebo  for 12 weeks. The participants and data collectors were blinded to the  treatment. Once all participants completed the study, the randomization  code was released.’’ | Yes (P2) | Yes (P8)  ‘’The  allocation to the NMN or placebo group was also managed by C&C  QUALITATIVE RESEARCH INSTITUTE INC until the end of the study. The  participants received oral supplementation of 250 mg of NMN (Mitsubishi  Corporation Life Sciences Limited, Tokyo, Japan) once daily or a placebo  for 12 weeks. The participants and data collectors were blinded to the  treatment. Once all participants completed the study, the randomization  code was released.’’ | Yes (P8)  ‘’The  allocation to the NMN or placebo group was also managed by C&C  QUALITATIVE RESEARCH INSTITUTE INC until the end of the study. The  participants received oral supplementation of 250 mg of NMN (Mitsubishi  Corporation Life Sciences Limited, Tokyo, Japan) once daily or a placebo  for 12 weeks. The participants and data collectors were blinded to the  treatment. Once all participants completed the study, the randomization  code was released.’’ | Yes (P8)  ‘’The  allocation to the NMN or placebo group was also managed by C&C  QUALITATIVE RESEARCH INSTITUTE INC until the end of the study. The  participants received oral supplementation of 250 mg of NMN (Mitsubishi  Corporation Life Sciences Limited, Tokyo, Japan) once daily or a placebo  for 12 weeks. The participants and data collectors were blinded to the  treatment. Once all participants completed the study, the randomization  code was released.’’ | No, (p2)  ‘’ The supplements (placebo or NMN) were supplied to each  group of participants at 0- and 6-week visits. However, after the  completion of the study, it came to light that at the 6-week visit,  11 participants each in the NMN and placebo groups received the  other supplement owing to an error made by the supplier.’’ | No, (p2)  ‘’ The supplements (placebo or NMN) were supplied to each  group of participants at 0- and 6-week visits. However, after the  completion of the study, it came to light that at the 6-week visit,  11 participants each in the NMN and placebo groups received the  other supplement owing to an error made by the supplier.’’ | Yes (P3-5) | Yes (P3-5) |
| Kim, 2022 | Yes (P3) ‘’ The inclusion criteria  had an average intake of caffeine more than 400 mg per day, (3) took supplements or  were: (1) independent mobility and active participation in the study, (2) participants without  ergy drinks containing NMN or niacin (Vitamin B3, nicotinamide, nicotinic’’ | YeS (P4)  ‘’divide 108 participants into  four groups using stratified randomization and permuted block randomization’’ | Yes (P4)  ‘’ To ensure the reliability  of the double-blind study, all data (personal information, group information, distribution  and collection of food and recording diary, and measurement results) were managed by  e-sports Co., Ltd. ll members remained completely anonymous to both participants  and researchers until key codes were revealed after 12 weeks at the completion of this  trial.’’ | Yes (P7)  including key characteristic of muscle mass. | Yes (P4)  ‘’ To ensure the reliability  of the double-blind study, all data (personal information, group information, distribution  and collection of food and recording diary, and measurement results) were managed by  e-sports Co., Ltd. ll members remained completely anonymous to both participants  and researchers until key codes were revealed after 12 weeks at the completion of this  trial.’’ | Yes (P4)  ‘’ To ensure the reliability  of the double-blind study, all data (personal information, group information, distribution  and collection of food and recording diary, and measurement results) were managed by  e-sports Co., Ltd. ll members remained completely anonymous to both participants  and researchers until key codes were revealed after 12 weeks at the completion of this  trial.’’ | Yes (P4)  ‘’ To ensure the reliability  of the double-blind study, all data (personal information, group information, distribution  and collection of food and recording diary, and measurement results) were managed by  e-sports Co., Ltd. ll members remained completely anonymous to both participants  and researchers until key codes were revealed after 12 weeks at the completion of this  trial.’’ | Yes (p3) | Yes (p3) | Yes (results) (p9) | Yes (p9) |
| Morifuji, 2024 | Yes (table 1, page 3) | Yes (page 6)  A total of 60 participants who met the selection criteria and did not violate the exclusion criteria were selected and randomly assigned into one of  two groups. | Yes (p4)  ‘’ Blinding throughout the  study was maintained by all parties except the study  substance allocation manager.’’ | Yes (p8) | Yes (p4)  ‘’ Blinding throughout the  study was maintained by all parties except the study  substance allocation manager.’’ | Yes (p4)  ‘’ Blinding throughout the  study was maintained by all parties except the study  substance allocation manager.’’ | Yes (p4)  ‘’ Blinding throughout the  study was maintained by all parties except the study  substance allocation manager.’’ | Yes (p7) | Yes (p7) | Yes (p6)  ‘’ The stepping test (sitting and standing) showed no  significant difference between the placebo and NMN  groups at either 4 or 12 weeks of intake (Table 4,  Supplementary Table S2).’’ | Yes (table 3, page 9) |
| Yoshino, 2021 | No | Yes (p2) ‘’ 12 were randomized to the placebo group and 13 to the NMN group’’ | No | Yes (p14) | Yes (p2) | No (no information) | No (no information) | No (no information on proportion of patients who completed study vs. total started.) | Yes (P9)  ‘’ Based on weekly pill counts, 99.6% of prescribed pills in  the placebo group and 100% of prescribed pills in the NMN group were taken by the  participants.’’ | No | Yes (p10) |
| Elhassan, 2019 | Yes (p16)  ‘’ All participants fulfilled the inclusion criteria including: male sex, age 70 – 80 years, BMI  20 – 30 kg/m2, able to discontinue aspirin’’ | Yes (p16)  ‘’ Participants were allocated to either NR or placebo. A randomization list was held by the clinical trials pharmacist at the clinical  research facility. The study investigators, nurses, and participants were all blinded to the intervention allocation during the trial.’’ | Yes (p16)  ‘’ Participants were allocated to either NR or placebo. A randomization list was held by the clinical trials pharmacist at the clinical  research facility. The study investigators, nurses, and participants were all blinded to the intervention allocation during the trial.’’ | Yes (Table s1) | Yes (p16)  ‘’ Participants were allocated to either NR or placebo. A randomization list was held by the clinical trials pharmacist at the clinical  research facility. The study investigators, nurses, and participants were all blinded to the intervention allocation during the trial.’’ | Yes (p16)  ‘’ Participants were allocated to either NR or placebo. A randomization list was held by the clinical trials pharmacist at the clinical  research facility. The study investigators, nurses, and participants were all blinded to the intervention allocation during the trial.’’ | Yes (p16)  ‘’ Participants were allocated to either NR or placebo. A randomization list was held by the clinical trials pharmacist at the clinical  research facility. The study investigators, nurses, and participants were all blinded to the intervention allocation during the trial.’’ | Yes (p3)  ‘’ All participants completed  the study visits (5 in total)’’ | Yes (p3)  ‘’ All participants completed  the study visits (5 in total) and assessments according to protocol (Figure S1).’’ | Yes, Figure 1, p4) | Yes, Figure 1, p4) |
| Martens, 2018 | Yes (P7) | Yes (P7) | Yes (p7)  ‘’ Randomization was performed by a member of the study team  not involved in the assessment of outcomes. The study participants and members of  the study team involved in the collection and analysis of outcomes were blinded to  the treatment condition.’’ | Yes (P3) | Yes (p7)  ‘’ Randomization was performed by a member of the study team  not involved in the assessment of outcomes. The study participants and members of  the study team involved in the collection and analysis of outcomes were blinded to  the treatment condition.’’ | Yes (p7)  ‘’ Randomization was performed by a member of the study team  not involved in the assessment of outcomes. The study participants and members of  the study team involved in the collection and analysis of outcomes were blinded to  the treatment condition.’’ | Yes (p7)  ‘’ Randomization was performed by a member of the study team  not involved in the assessment of outcomes. The study participants and members of  the study team involved in the collection and analysis of outcomes were blinded to  the treatment condition.’’ | No (Figure 1, page 2)  24/30 completed and assessed | Yes (P2) | Yes (p3-4) | Yes (p3-4) |
| McDermott, 2024 | Yes (p5) | Yes (p5) | No | Yes | Yes (p5) | Yes (p6) | Yes (p6) | Yes (Figure 1, page 2) | Yes (Results, page 2) | Yes (page 2)  ‘’ Compared to placebo, NR improved 6-min walk by 17.6 meters (90%  CI: + 1.77, +∞  , P = 0.08) at 6-month follow-up,’’ | Yes (page 2)  ‘’ Compared to placebo, NR improved 6-min walk by 17.6 meters (90%  CI: + 1.77, +∞  , P = 0.08) at 6-month follow-up,’’ |

| Orr, 2024 | Yes (p3) | Yes (p4)  ‘’ This was a 10 week, double-blind, randomized,  placebo-controlled study (Fig. 1). Twenty (20)  MCI subjects were randomized (1:1) to receive  either placebo (n=10) or nicotinamide riboside  (n=10) (NR, NIAGEN, Chroma Dex Inc.) two 250  mg twice a day’’ | No | Yes (table 1, page 7) | Yes (p4)  ‘’ This was a 10 week, double-blind, randomized,  placebo-controlled study (Fig. 1). Twenty (20)  MCI subjects were randomized (1:1) to receive  either placebo (n=10) or nicotinamide riboside  (n=10) (NR, NIAGEN, Chroma Dex Inc.) two 250  mg twice a day, orally, for a total of 1 g daily as  tolerated.’’ | Yes (p4)  ‘’ to receive a new bottle of capsules  and to discuss any issues with tolerability or treatment-  emergent adverse events (AEs) with a member of the  research team who was not involved in data collection  or analysis in order to ensure blinding of the investigators.’’ | Yes (p4)  ‘’ to receive a new bottle of capsules  and to discuss any issues with tolerability or treatment-  emergent adverse events (AEs) with a member of the  research team who was not involved in data collection  or analysis in order to ensure blinding of the investigators.’’ | Yes (page 7) | Yes (page 7) | Yes (page 5, page 7) | Yes (table 2, page 10) |
| --- | --- | --- | --- | --- | --- | --- | --- | --- | --- | --- | --- |
| Wang, 2022 | Yes  (p3)  ‘’ The 30 randomized participants met all prespecified inclusion criteria of: 1) HF with reduced  ejection fraction (LV ejection fraction #40%, as  determined by transthoracic echocardiogram)..’’ | Yes (p4)  ‘’ Using a randomization table provided by the trial  biostatistician, the University of Washington Investigational Drug Service assigned trial IDs and dispensed  trial medications. Participants were randomized to NR  or matching placebo at a 2:1 allocation ratio.’’ | Yes (p4)  ‘’ In this double-blind study, participants and study  investigators or personnel were blinded to treatment  assignment, as well as to blood NR and NAD levels.  Using a randomization table provided by the trial  biostatistician, the University of Washington Investigational Drug Service assigned trial IDs and dispensed  trial medications. Participants were randomized to NR  or matching placebo at a 2:1 allocation ratio.’’ | Yes (p6) | Yes (p4)  ‘’ In this double-blind study, participants and study  investigators or personnel were blinded to treatment  assignment, as well as to blood NR and NAD levels.  Using a randomization table provided by the trial  biostatistician, the University of Washington Investigational Drug Service assigned trial IDs and dispensed  trial medications. Participants were randomized to NR  or matching placebo at a 2:1 allocation ratio.’’ | ’ In this double-blind study, participants and study  investigators or personnel were blinded to treatment  assignment, as well as to blood NR and NAD levels.  Using a randomization table provided by the trial  biostatistician, the University of Washington Investigational Drug Service assigned trial IDs and dispensed  trial medications. Participants were randomized to NR  or matching placebo at a 2:1 allocation ratio.’’ | ’ In this double-blind study, participants and study  investigators or personnel were blinded to treatment  assignment, as well as to blood NR and NAD levels.  Using a randomization table provided by the trial  biostatistician, the University of Washington Investigational Drug Service assigned trial IDs and dispensed  trial medications. Participants were randomized to NR  or matching placebo at a 2:1 allocation ratio.’’ | Yes (p3)  30/33 completed study with assessments available | Yes (p3)  30/33 completed study with assessments available | Yes (p7,10; figure 3) | Yes (p7,10; figure 3) |

Q1: eligibility criteria were specified
Q2: subjects were randomly allocated to groups (in a crossover study, subjects were randomly allocated an order in which treatments were received)
Q3: allocation was concealed
Q4: the groups were similar at baseline regarding the most important prognostic indicators
Q5: there was blinding of all subjects
Q6: there was blinding of all therapists who administered the therapy
Q7: there was blinding of all assessors who measured at least one key outcome
Q8: measures of at least one key outcome were obtained from more than 85% of the subjects initially allocated to groups
Q9: all subjects for whom outcome measures were available received the treatment or control condition as allocated or, where this was not the case, data for at least one key outcome was analysed by “intention to treat”
Q10: the results of between-group statistical comparisons are reported for at least one key outcome
Q11: the study provides both point measures and measures of variability for at least one key outcome
